# Prediction of the local treatment outcome in patients with oropharyngeal squamous cell carcinoma using deep learning analysis of pretreatment FDG-PET images

**DOI:** 10.1186/s12885-021-08599-6

**Published:** 2021-08-06

**Authors:** Noriyuki Fujima, V. Carlota Andreu-Arasa, Sara K. Meibom, Gustavo A. Mercier, Minh Tam Truong, Kenji Hirata, Koichi Yasuda, Satoshi Kano, Akihiro Homma, Kohsuke Kudo, Osamu Sakai

**Affiliations:** 1 Departments of Radiology, Boston University School of Medicine, One Boston Medical Center Place, 3rd Floor, 820 Harrison Avenue, Boston, MA 02118 USA; 2 Research Center for Cooperative Projects, Hokkaido University Graduate School of Medicine, kita 15, nishi 7, kita-ku, Sapporo, Hokkaido 060-8638 Japan; 3 Departments of Radiation Oncology, Boston Medical Center, Boston University School of Medicine, One Boston Medical Center Place, Boston, MA 02118 USA; 4 Departments of Diagnostic Imaging, Hokkaido University Graduate School of Medicine, kita 15, nishi 7, kita-ku, Sapporo, Hokkaido 060-8638 Japan; 5 Departments of Radiation Medicine, Hokkaido University Graduate School of Medicine, kita 15, nishi 7, kita-ku, Sapporo, Hokkaido 060-8638 Japan; 6 Otolaryngology-Head and Neck Surgery, Hokkaido University Graduate School of Medicine, kita 15, nishi 7, kita-ku, Sapporo, Hokkaido 060-8638 Japan; 7 The Global Station for Quantum Medical Science and Engineering, Global Institution for collaborative research and education, Sapporo, Hokkaido 060-0808 Japan

**Keywords:** Deep learning, Oropharyngeal squamous cell carcinoma, FDG-PET, Treatment outcome

## Abstract

**Background:**

This study aimed to assess the utility of deep learning analysis using pretreatment FDG-PET images to predict local treatment outcome in oropharyngeal squamous cell carcinoma (OPSCC) patients.

**Methods:**

One hundred fifty-four OPSCC patients who received pretreatment FDG-PET were included and divided into training (*n* = 102) and test (*n* = 52) sets. The diagnosis of local failure and local progression-free survival (PFS) rates were obtained from patient medical records. In deep learning analyses, axial and coronal images were assessed by three different architectures (AlexNet, GoogLeNET, and ResNet). In the training set, FDG-PET images were analyzed after the data augmentation process for the diagnostic model creation. A multivariate clinical model was also created using a binomial logistic regression model from a patient’s clinical characteristics. The test data set was subsequently analyzed for confirmation of diagnostic accuracy. Assessment of local PFS rates was also performed.

**Results:**

Training sessions were successfully performed with an accuracy of 74–89%. ROC curve analyses revealed an AUC of 0.61–0.85 by the deep learning model in the test set, whereas it was 0.62 by T-stage, 0.59 by clinical stage, and 0.74 by a multivariate clinical model. The highest AUC (0.85) was obtained with deep learning analysis of ResNet architecture. Cox proportional hazards regression analysis revealed deep learning-based classification by a multivariate clinical model (*P* < .05), and ResNet (*P* < .001) was a significant predictor of the treatment outcome. In the Kaplan-Meier analysis, the deep learning-based classification divided the patient’s local PFS rate better than the T-stage, clinical stage, and a multivariate clinical model.

**Conclusions:**

Deep learning-based diagnostic model with FDG-PET images indicated its possibility to predict local treatment outcomes in OPSCCs.

**Supplementary Information:**

The online version contains supplementary material available at 10.1186/s12885-021-08599-6.

## Background

In patients with oropharyngeal squamous cell carcinoma (OPSCC), the treatment method is generally selected based on patient characteristics and TNM (tumor, node, metastasis) staging. Most patients with advanced OPSCC undergo nonsurgical treatments such as chemotherapy, radiotherapy, and combinations of these [1]. When receiving such nonsurgical therapies, the prediction of treatment outcome helps optimize patient management because it allows for additional treatment and the determination of a follow-up strategy. For pretreatment evaluation for OPSCCs, TNM staging has been widely used as the standard method that classifies each primary site based on the morphological information and extent of the tumor. TNM classification was reported to correlate with the treatment outcome and patient prognosis [2, 3], however this method cannot completely predict the treatment outcome due to overlap between good and poor prognosis patients. In a recent report investigated the prognosis in patients with OPSCC, around 30% of patients with early T-stage (T1 and T2) resulted in poor treatment outcome, whereas approximately 40% of patients with advanced T-stage (T3 and T4) resulted in good treatment outcome [4]. Such overlap is considered one of the major limitations in the current staging system. To add other treatment options (e.g., induction chemotherapy, additional surgical resection, etc.), or to receive the de-intensified treatment method might be acceptable if a more accurate predictive model is available; this may result in personalized treatment planning.

FDG-PET depicts a tumor’s glucose metabolism, one of the important factors that reflect tumor functional information. Currently, FDG-PET/CT scanners are widely available for clinical use. Several studies have investigated FDG-PET parameters in OPSCC patients to predict treatment outcomes as prognostic factors, mainly investigating the maximum standardized uptake value (SUVmax), mean standardized uptake value (SUVmean), metabolic tumor volume (MTV), and total lesion glycolysis (TLG) [5–7]. Moreover, tumor quantitative morphological data and intra-tumoral heterogeneity obtained by FDG-PET imaging were reported to be useful in predicting prognosis in several studies [8–11]. These previous reports indicate FDG-PET imaging has information suitable to predict patient prognosis. Recently, artificial intelligence-based diagnosis using deep learning techniques has been introduced in medical image analysis. The deep learning-based medical image analysis enables identifying features and textures inherent in the original images; the deep learning technique has a potential to accomplish the image analysis with high diagnostic performance [12]. However, few studies investigated the usefulness of deep learning analysis in head and neck cancer imaging to predict local treatment outcomes [13, 14]. To our knowledge, there is no previous study to predict the local treatment outcome of OPSCCs by deep learning techniques using the FDG-PET imaging dataset.

This study investigated the value of deep learning analysis of FDG-PET images to predict the local treatment outcome in OPSCC patients who received nonsurgical treatment.

## Methods

### Patients

This retrospective study’s protocol was approved by the institutional review boards at the two participating institutions (Institution A and Institution B), and written informed consent from the patient was waived. One hundred sixty-seven patients with histopathologically proven OPSCC who underwent pretreatment FDG-PET/CT and chemoradiation treatment with a radiation dose of 65–70 Gy with curative intent from January 2007 to August 2017 were enrolled. However, patients with 1) severe motion artifact that seriously affected the image quality of the primary lesion (*n* = 2) and 2) primary lesions that were too small with the largest diameter less than 1.5 cm (*n* = 11) were excluded. Finally, 154 patients were considered eligible for this study. We analyzed FDG-PET/CT image datasets of patients enrolled at Institution A (102 patients) for model development as a training set and patients enrolled at Institution B (52 patients) for the model validation as a test set. All patients were treated with chemoradiation therapy with curative intent; the treatment regimen was composed of systemic platinum-based chemotherapy with concurrent radiotherapy of 65–70 Gy. No patient underwent surgery prior to chemoradiation therapy. As an additional treatment option, 25 patients in the training set and 19 patients in the test set received 1–5 courses of induction chemotherapy before the definitive chemoradiation therapy after pretreatment FDG-PET/CT. For the management of residual nodal diseases after definitive chemoradiation therapy, eleven patients in the training set and five patients in the test set underwent additional neck dissection for the resectable nodal disease. Essential patients’ characteristics are summarized in Table 1.

**Table 1:** Patient characteristics

	Total (*n* = 154)	Training cohort (*n* = 102)	Test cohort (*n* = 52)	*P*-value
Age				
Range	39–82	41–81	39–82	0.24
Median	60	59	62	
Average	59.8	59.1	61.3	
Sex				
Male	128 (83%)	82 (80%)	46 (88%)	0.21
Female	26 (17%)	20 (20%)	6 (12%)	
HPV status				
Positive	46 (30%)	35 (34%)	11 (21%)	0.09
Negative	33 (21%)	25 (25%)	8 (15%)	
Unknown	75 (49%)	42 (41%)	33 (63%)	
T-stage				
T1	16 (10%)	12 (12%)	4 (8%)	0.32
T2	36 (23%)	21 (21%)	15 (29%)	
T3	46 (30%)	27 (26%)	19 (36%)	
T4a	39 (26%)	30 (29%)	9 (17%)	
T4b	17 (11%)	12 (12%)	5 (10%)	
N-stage				
N0	14 (9%)	5 (5%)	9 (17%)	0.16
N1	19 (12%)	14 (14%)	5 (10%)	
N2a	14 (9%)	8 (8%)	6 (12%)	
N2b	58 (38%)	38 (37%)	20 (38%)	
N2c	42 (27%)	32 (31%)	10 (19%)	
N3	7 (5%)	5 (5%)	2 (4%)	
Clinical Stage				
I	2 (1%)	2 (2%)	0	0.07
II	15 (10%)	12 (12%)	3 (6%)	
III	30 (19%)	24 (24%)	8 (15%)	
IVa	88 (57%)	51 (50%)	35 (67%)	
IVb	19 (12%)	13 (13%)	6 (12%)	
IVc	0	0	0	
Induction chemotherapy				
administered	44 (29%)	25 (25%)	19 (36%)	0.09
not administered	110 (71%)	77 (75%)	33 (64%)	

*P*-value was obtained by comparing patients’ characteristics between training and test cohorts using Mann–Whitney U test

### Clinical assessment

Clinical and radiological follow-up at least 2 years was performed after treatment to determine the final diagnosis (i.e., local control or failure) in all patients. If residual and/or recurrent tumor was suspected in the follow-up period by direct visualization or radiological image findings (e.g., mass-like lesion development at the post-treatment site), surgical biopsy or resection was conducted for the confirmation of the presence of residual or recurrent tumor histopathologically. If the patient didn’t consent to the surgical procedure, an observation was carefully continued. Local failure was defined by the histopathological confirmation of SCC by biopsy or surgical resection or the observation of a clearly developed or enlarged mass lesion at the post-treatment site. Local control was defined by histopathological confirmation of the absence of SCC by surgical resection (not by biopsy) or no enlargement or abnormality of soft tissue at the post-treatment site throughout the follow-up period. In addition, we determined the local progression-free survival (PFS) duration of all patients of the test cohort during the individual follow-up periods after curative treatment using the patients’ medical records.

### Image acquisition

From the pretreatment FDG-PET/CT dataset, axial and coronal FDG-PET images were used for evaluation. FDG-PET/CT imaging was performed on specific PET/CT scanners (Discovery STE 16, GE Healthcare, Milwaukee, Wisconsin) in the training cohort at Institution A, and another specific PET/CT scanner (Asahi-Siemens Medical Technologies, Tokyo) in the test cohort at Institution B. At all scanners, all FDG-PET raw images were obtained with an axial-base acquisition. The following basic parameters were used: the FOV of 30 cm, the matrix size of 128 × 128 and the slice thickness of 3.3 mm for the training cohort, and the FOV of 21.6 cm, the matrix of 168 × 168, and the slice thickness of 2.0 mm for the test cohort. Coronal images were thereafter reconstructed from FDG-PET raw data. Patients were injected with an average of 9.98 mCi of ^18^F- FDG, and it was incubated for approximately 60 min. The SUV was defined as the tissue concentration of radioactivity (kBq/mL) divided by the injected dose per body weight (kBq/g).

### Image processing

**Fig. 1:**
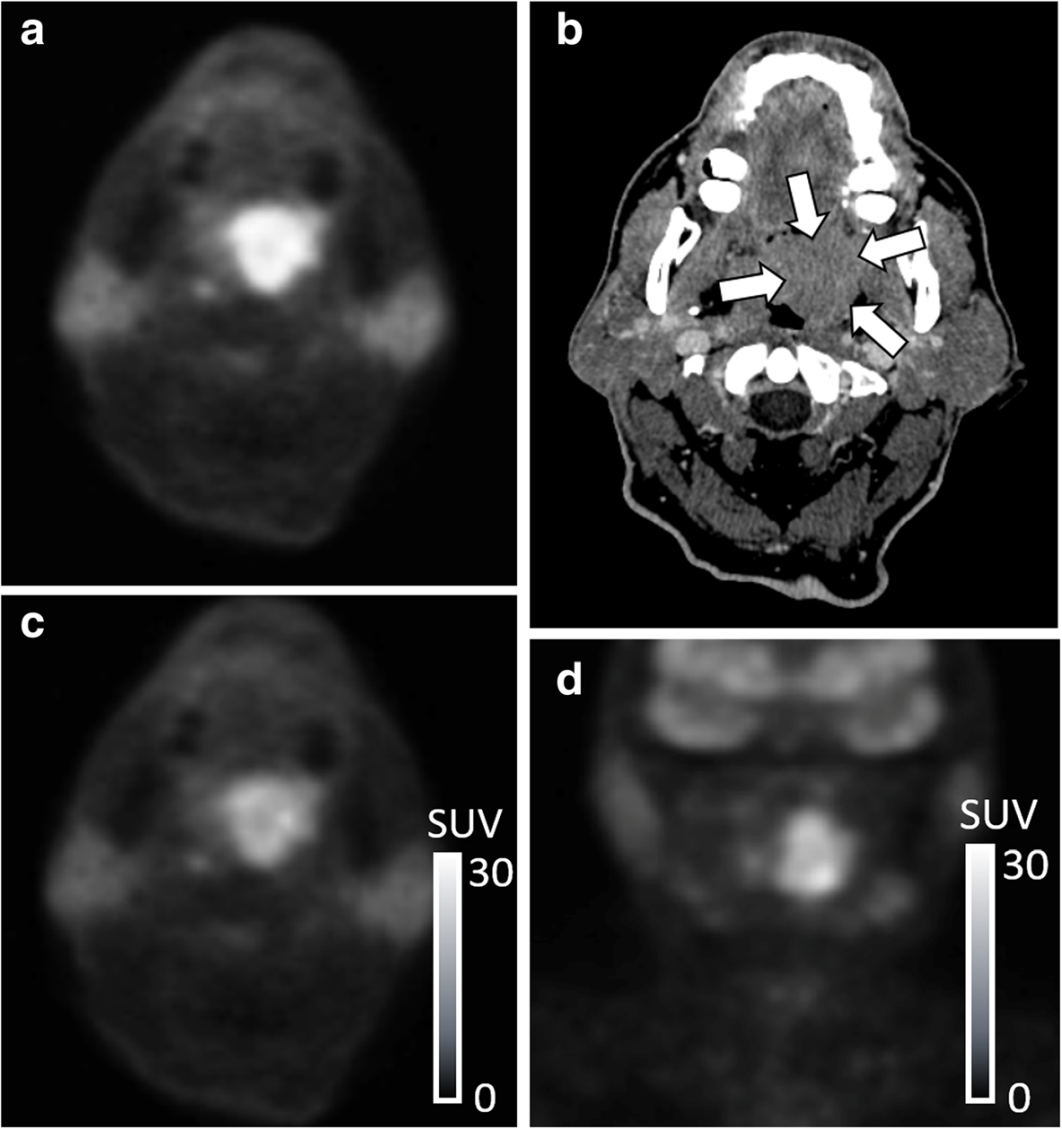
Processes of FGD-PET image for deep learning analysis. The primary tumor site on FDG-PET images (**A**) was carefully identified with the reference of CT images (**B**, *arrow*). Axial 2D FDG-PET image in which the largest area of the tumor was depicted was selected for analysis, and then the FDG-PET image was outputted with the fixed grayscale level of black (SUV = 0) to white (SUV = 30) (**C**). Coronal FDG-PET image was also selected and outputted in the same manner as axial image selection using a coronal reconstructed image dataset (**D**)

**Fig. 2:**
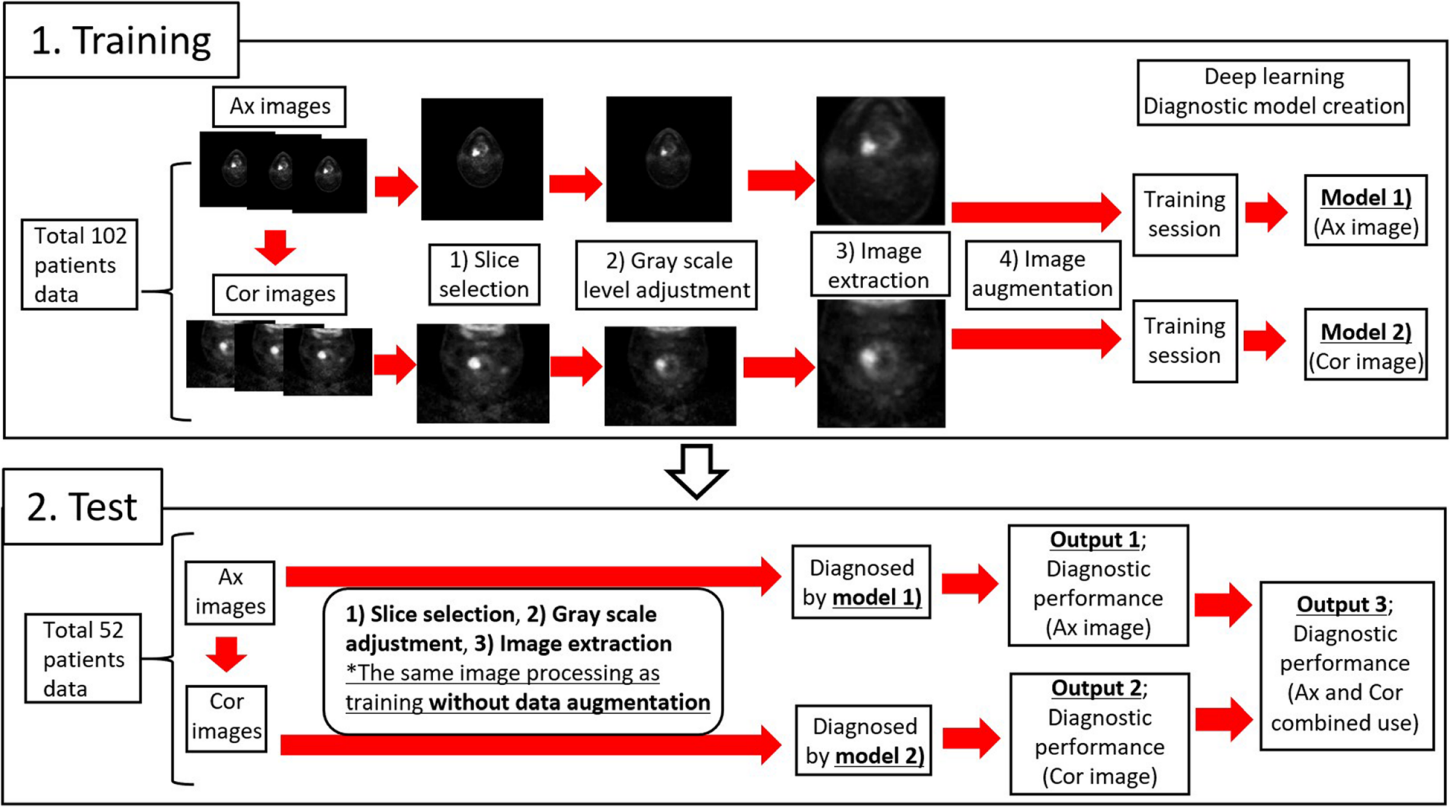
Overview of Deep learning workflow. The workflow of both the training and test processes was presented. In the training session, image processing was performed as follows: 1) slice selection; the specific slice which included the largest tumor area was selected, 2) grayscale adjustment; setting the pixel with its SUV of 0 (=lower limit) and its SUV of 30 (=upper limit), 3) image conversion from the DICOM to JPEG picture data, 4) data augmentation; an additional 23 images were generated from each image. These images were fed into the deep learning training session, thereafter, the axial and coronal image-based diagnostic model was respectively created. In the test session, we performed the same imaging processing without data augmentation in original images of test cohorts. These processed images were classified by the diagnostic model created in the training session. Finally, diagnostic performance by axial, coronal, and axial-coronal combination use was respectively obtained

First, the location of FDG uptake in the primary site was carefully identified on both axial and coronal FDG-PET images. Simultaneously acquired CT images were used as the reference for the identification of the primary site. Each tumor was delineated with a threshold SUV value; the threshold was set 42% of the maximum SUV in the whole tumor (i.e., SUVmax) to define the tumor area [15]. In all slices with the tumor FDG uptake, a specific slice that included the largest tumor area (i.e., the largest number of pixels) was selected in axial and coronal planes and further analyzed. All selected images were converted from the DICOM to Joint Photographic Experts Group (JPEG) picture data; the grayscale level was set so that the pixel with non-FDG uptake (i.e., SUV = 0) becomes black (lower limit) and the pixel with its SUV of 30 becomes white (upper limit) [16]. These processes are illustrated in Fig. 1. After the image selection and conversion from DICOM to JPEG picture data, all these images were cropped into 20 × 20 cm FOV, eliminating peripheral background areas, thereafter an image augmentation process was performed. The process was based on a random change in image size, random image rotation, and random pixel shift of image location along x- and y-directions [17]; a total of 24 images were finally generated for each image. The training image cohort consisted of 2448 images from 102 patients (102 × 24 images) in both axial and coronal images. In addition, 52 images of the respective axial and coronal axis from 52 patients in the test cohort were generated with the aforementioned image processing in the training dataset but without the data augmentation process. An overview of the deep learning workflow is illustrated in Fig. 2.

### Deep learning analysis

The deep learning analysis using the training dataset was performed to create a diagnostic model for the discrimination between local control and local failure cases. Axial and coronal images were independently used as input images. We performed all analyses using MATLAB ver. 2019a (MathWorks, Natick, MA). As an architecture for the convolutional neural network (CNN) analysis, deep learning models of AlexNet, GoogLeNet (GoogLeNet Inception v3), and ResNet (ResNet-101), all of which were trained on an image database (i.e., ImageNet database: http://image-net.org/index), was respectively used. Previous reports described the detail of the architectures of AlexNet [18], GoogLeNet [19], and ResNet [20]. Hyperparameters were set as the mini-batch size of 128, the max epoch of 500. We used the transfer learning to adjust the image features in the ImageNet database to account for our imaging data, including local control and failure OPSCCs scans. Fine-tuning of the CNN over FDG-PET images with treatment control and failure was performed only on the fully connected layer, whereas other prior layers were fixed. Finally, respective diagnostic models were created from training dataset using the three deep learning architectures of AlexNet, GoogleNet, and ResNet. From two diagnostic models created by axial and coronal images per an architecture, we defined the diagnostic scores obtained by deep learning analyses as follows; 1) axial image only used model; diagnosed as local control (low-risk group, score = 0) and diagnosed as a local failure (high-risk group, score = 1), 2) coronal image only used model; diagnosed as local control (low-risk group, score = 0) and diagnosed as a local failure (high-risk group, score = 1), 3) axial and coronal images combined model; diagnosed local control in both axial and coronal images (low-risk group, score = 0), diagnosed local control either of axial or coronal image (intermediate group, score = 1) and diagnosed local failure both of axial and coronal images (high-risk group, score = 2). In the training set, these three types (axial images only, coronal images only, and the axial and coronal images combined model) of a scoring system based on deep learning diagnostic model from three types of deep learning architectures for the classification were respectively created; total nine classification models were obtained. Thereafter, these classification models were assessed against the test dataset to obtain the optimal cut-off score in each model and to determine its diagnostic performance. In this process, the optimal cut-off value for axial or coronal images only used models were automatically determined because these models were binary output (score 0 or 1), whereas optimal cut-off value in axial and coronal images combined model was determined by statistical analytical method (see below Statistical analysis) because this model was based on 3 grading system (score = 0, 1 and 2).

In addition, we created a multivariate clinical model using the patient characteristics of T-stage, clinical stage, age, the presence of induction chemotherapy, and the HPV status from training cohorts. Based on patient local treatment outcome in training cohorts, a binomial logistic regression model was used to fit the binary treatment outcome. Each regression coefficient for each patient characteristic was determined to create the regression equation.

### Statistical analysis

Firstly, essential patients’ characteristics were compared using Mann–Whitney U test between training and test cohorts.

After the creation of both diagnostic models using deep learning analysis and multivariate clinical model with training cohorts, we first conducted a receiver operating characteristic (ROC) curve analysis using three types of deep learning risk classification obtained by analyses with AlexNet, GoogleNet, and ResNet architecture to assess the predictive power to discriminate between local control and failure with test cohorts. The T-stage (1, 2, 3, 4a, and 4b), clinical stage (I, II, III, IVa, IVb, and IVc), and the multivariate clinical model (continuous variable) were also assessed by ROC curve analysis. In the ROC curve analysis, we calculated the area under curve (AUC) value and the optimal cut-off value using the Youden index in the division of patients with local control and failure within the whole follow-up period. We also calculated the sensitivity, specificity, positive predictive value (PPV), negative predictive values (NPV), and accuracy.

The deep learning model’s diagnostic performance with the highest AUC was subsequently analyzed to compare to T-stage, clinical stage, and a multivariate clinical model. First, this highest AUC value provided by the specific deep learning model was compared to that in T-stage and clinical stage, respectively, using the chi-square test. A multivariate Cox proportional hazards regression analysis was also performed among these four indexes (i.e., the specific deep learning model with the highest AUC, T-stage, clinical stage, and a multivariate clinical model) to predict the outcomes, including the time point at which local failure was determined with test cohorts. Next, using optimal cut-off value, we performed a Kaplan-Meier curve analysis to assess local PFS using the deep learning model with the highest AUC, T-stage, clinical stage, and multivariate clinical model with test cohorts. In the Kaplan-Meier curve analysis, we divided the patients into two groups using the cut-off value obtained in the ROC curve analysis. As subgroup analysis to focus on the HPV status, patients whose HPV status was available in test set cohorts were picked up and subsequently analyzed; the Kaplan-Meier curve analysis was performed to assess local PFS rates using the aforementioned deep learning model with the highest AUC and the HPV status (HPV positive versus negative), respectively. In addition, as the more detailed subgroup analysis, we further divided patients whose HPV status was available in test set cohorts into HPV positive and negative groups. Thereafter, in the HPV positive patient group, the Kaplan-Meier curve analysis was performed to assess local PFS rates using the aforementioned deep learning model with the highest AUC and a multivariate clinical model. The same analysis was also performed in the HPV negative patient group. The local PFS rates in all Kaplan-Meier curve analyses were assessed using the log-rank test.

To assess the robustness in creating deep learning diagnostic models when using different patient cohorts for training and test session, we performed additional analysis by switching patients’ cohorts between training and test session. First, patient cohorts in institution A (*n* = 102) were randomly divided into two groups; group A-1 (51 patients) and group A-2 (remained 51 patients). We performed a training session and created a diagnostic model using the combination of group A-1 and patients in institution B (total 103 patients) with the aforementioned deep learning model with the highest AUC. Thereafter, a test session was performed using patient group A-2. We also performed analysis using another combination of patient cohorts with a training session (combination of the group A-2 and patients in institution B, total 103 patients) and test session (group A-1, 51 patients). In each data of test session, sensitivity, specificity, PPV, NPV, and accuracy were calculated. Kaplan-Meier curve analysis was also performed.

*P*-values < 0.05 were considered significant in these analyses. SPSS software (IBM, Armonk, NY) was used for all statistical analyses.

## Results

The training cohort included 102 patients with local control in 69 and local failure in 33 patients as per the review of medical records in their follow-up period. The test cohort included 52 patients with local control in 35 and local failure in 17 patients. Any essential patients’ characteristics were not significantly different between training and test cohorts (Table 1).

The deep learning algorithm achieved optimal diagnostic performance after the training session with three deep learning architecture types. Diagnostic accuracy revealed after the final epoch in the training session were as follows; AlexNet with axial image (accuracy: 0.77) and coronal image (accuracy: 0.74), GoogLeNet with axial image (accuracy: 0.87) and coronal image (accuracy: 0.84), and ResNet with axial image (accuracy: 0.89) and coronal image (accuracy: 0.84), respectively. In addition, a multivariate clinical model was determined using a binomial logistic regression model using training cohorts. The standardized partial regression coefficient of each variable was as follows; T-stage 0.55, clinical stage 0.31, age 0.62, the presence of induction chemotherapy 0.06, and the HPV status 0.23.

**Fig. 3:**
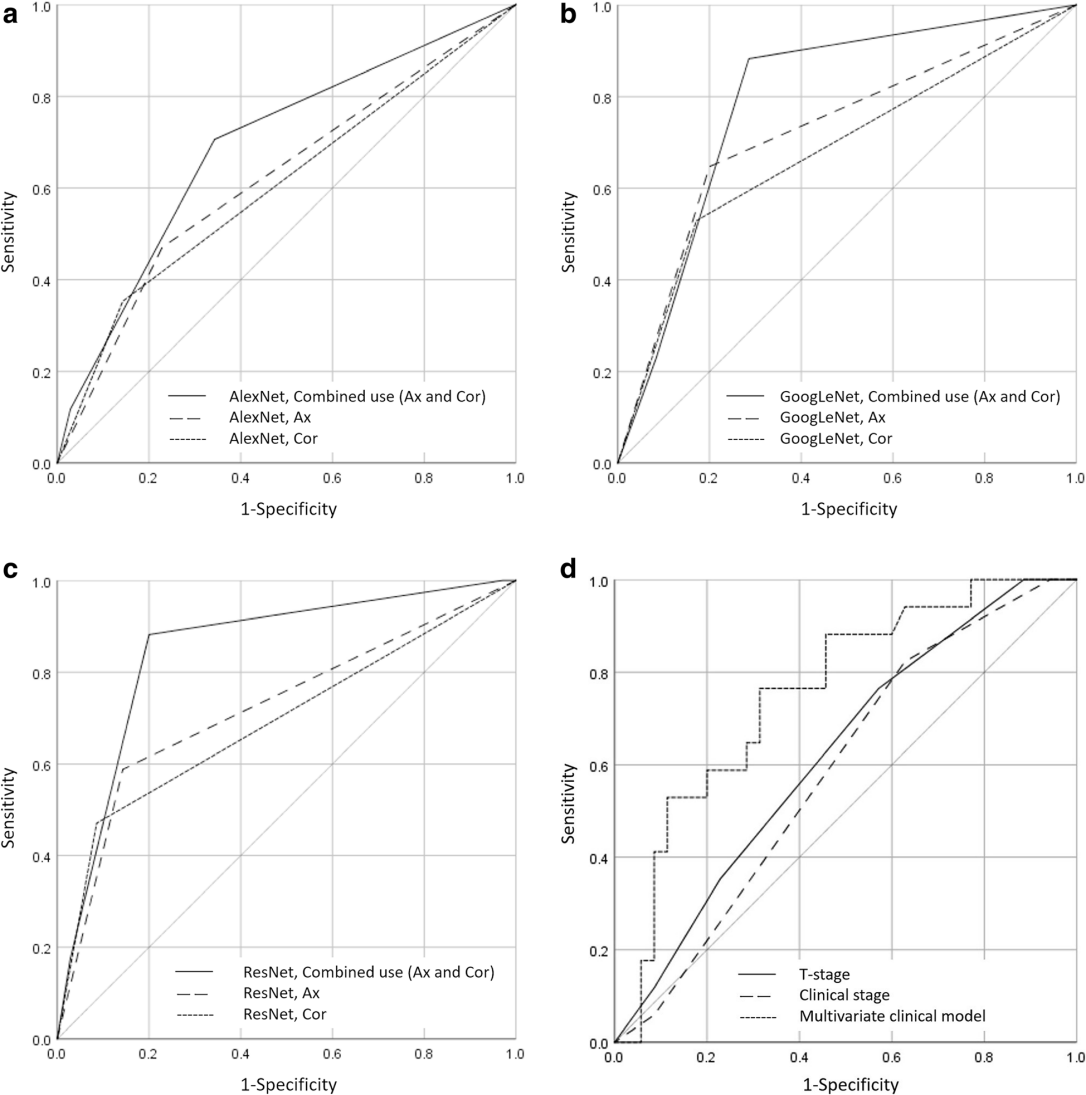
Results of ROC curve analyses. ROC curves obtained by deep learning analysis with axial image, coronal image, and these two images combination models in AlexNet (**A**), GoogLeNet (**B**), and ResNet (**C**) to determine the treatment outcome were presented. The ROC curve obtained by T-stage, clinical stage, and a multivariate clinical model was also shown (**D**). The highest AUC was obtained with the two images combination model in ResNet (AUC = 0.85)

Using the test set, a total of 12 ROC curve analyses were performed in axial, coronal, and a combination of axial and coronal images with three architecture of deep learning analysis and the T-stage, clinical stage, and a multivariate clinical model classification. The highest AUC (=0.85) was obtained using the combination use of axial and coronal images with deep learning analysis of ResNet architecture. All ROC curves are shown in Fig. 3. From the optimal cut-off values determined by the ROC curve, diagnostic test performance was computed. The highest diagnostic accuracy (=0.83) was also seen with the combined use of axial and coronal images with the ResNet architecture with the specific division setting between the low-risk group (=prediction of local control, score = 0) and intermediate/high-risk group (=predicted local failure, score = 1 or 2). All diagnostic parameters for all discrimination models are shown in Table. 2.

**Table 2:** Diagnostic performance obtained by ROC curve analyses

Classifier	AUC	Sensitivity	Specificity	PPV	NPV	Accuracy
DL AlexNet						
Ax	0.62 (0.45, 0.79)	0.47	0.77	0.5	0.75	0.67
Cor	0.61 (0.43, 0.77)	0.35	0.86	0.55	0.73	0.69
Ax and Cor combined	0.69 (0.54, 0.84)	0.71	0.69	0.52	0.83	0.69
DL GoogLeNet						
Ax	0.72 (0.57, 0.88)	0.65	0.8	0.61	0.82	0.75
Cor	0.68 (0.52, 0.84)	0.53	0.83	0.6	0.78	0.73
Ax and Cor combined	0.8 (0.67, 0.92)	0.94	0.71	0.62	0.96	0.79
DL ResNet						
Ax	0.72 (0.56, 0.88)	0.59	0.86	0.67	0.81	0.77
Cor	0.69 (0.53, 0.86)	0.47	0.91	0.73	0.78	0.77
Ax and Cor combined	0.85 (0.73, 0.96)	0.88	0.8	0.68	0.93	0.83
Conventional method						
T-stage	0.62 (0.47, 0.78)	0.76	0.43	0.4	0.79	0.54
Clinical stage	0.59 (0.43, 0.74)	0.82	0.4	0.4	0.82	0.54
Multivariate clinical model	0.74 (0.61, 0.89)	0.76	0.62	0.5	0.84	0.67

Data in parentheses are 95% confidence intervals, *AUC* Area under curve, *DL* Deep learning, *Ax* Axial, *Cor* Coronal, *PPV* Positive predictive value, *NPV* Negative predictive value

In the comparison of AUCs between ResNet with combination use of axial and coronal images and conventional methods (i.e., T-stage, clinical stage, and a multivariate clinical model), AUC obtained by ResNet was significantly higher than that by T-stage (*P* < .01) and was also significantly higher than that by clinical stage (*P* < .001). In contrast, AUC obtained by ResNet was higher than a multivariate clinical model. However, statistical significance was not observed (*P* = 0.18).

A multivariate Cox proportional hazards regression analysis revealed that deep learning-based classification with axial and coronal combination model in ResNet was a significant predictor of the treatment outcome (*P* < .001, Hazard ratio 3.69, 95% confidence intervals 1.72–7.88). A multivariate clinical model was also revealed significant (*P* < .05, Hazard ratio 1.74, 95% confidence intervals 1.09–2.87). In contrast, T-stage (*P* = 0.29, Hazard ratio 1.24, 95% confidence intervals 0.81–1.68) and clinical stage (*P* = 0.48, Hazard ratio 0.72, 95% confidence intervals 0.24–1.92) was not significant respectively.

**Fig. 4:**
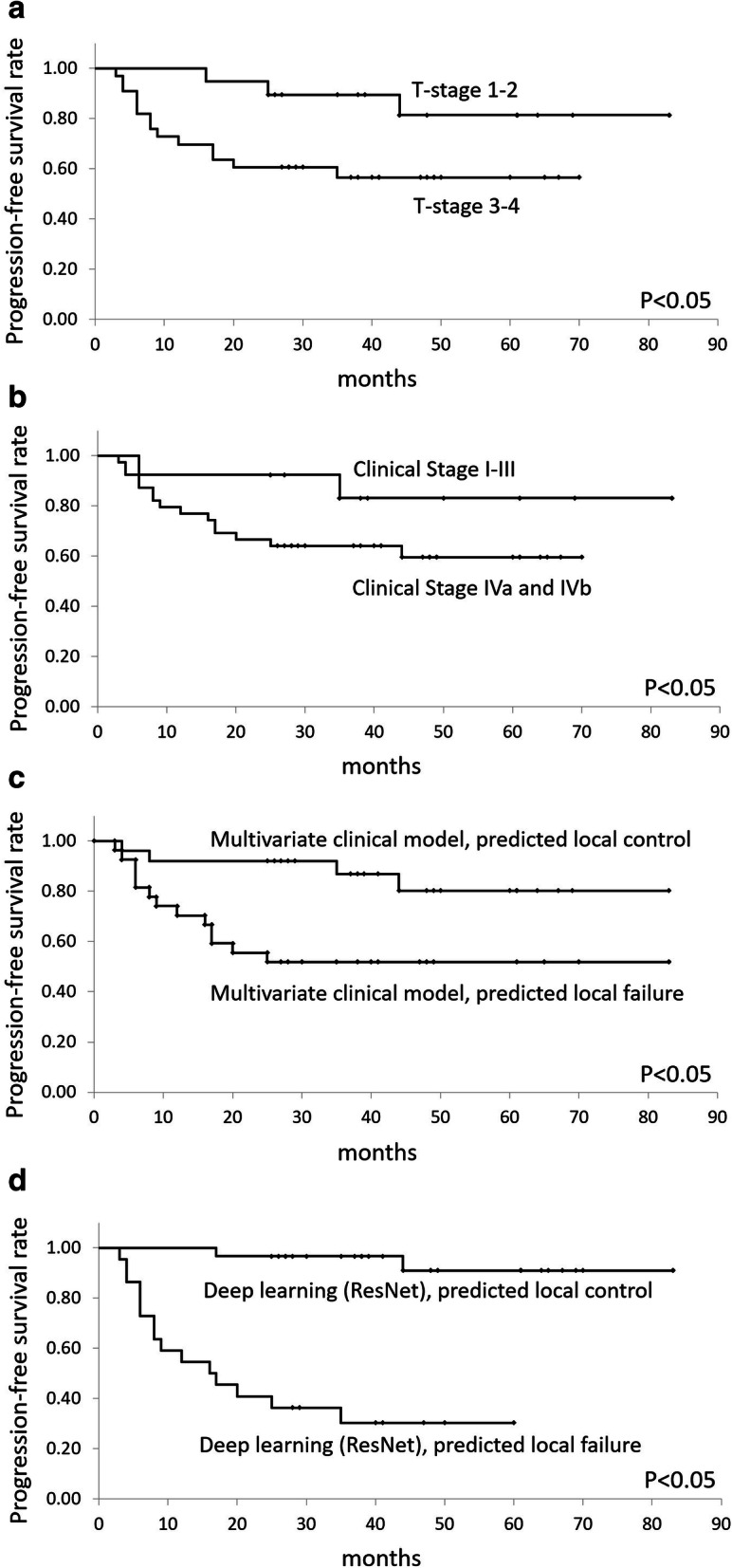
Results of the Kaplan-Meier analyses. The local PFS rate was significantly greater in lower T-stage (**A**), lower clinical stage (**B**), local control predicted group by a multivariate clinical model (**C**), and low risk of deep learning-based classification with axial and coronal images combination model in ResNet (**D**) (*P* < .05, respectively). The local PFS rate could be divided more clearly in a deep learning-based classification model

In the Kaplan-Meier analysis with the respective cut-off value determined by ROC curve analysis, the local PFS rate was significantly greater in all the group of patients with lower T-stage (T1 and T2), lower clinical stage (I, II, and III), local control predicted group by a multivariate clinical model, and local control predicted group by deep learning-based classification with axial and coronal combination model in ResNet (*P* < .05, respectively). The local PFS rate could be divided more clearly in the Kaplan-Meier curve using the deep learning-based classification model. Analysis of the Kaplan-Meier curves is summarized in Fig. 4.

**Fig. 5:**
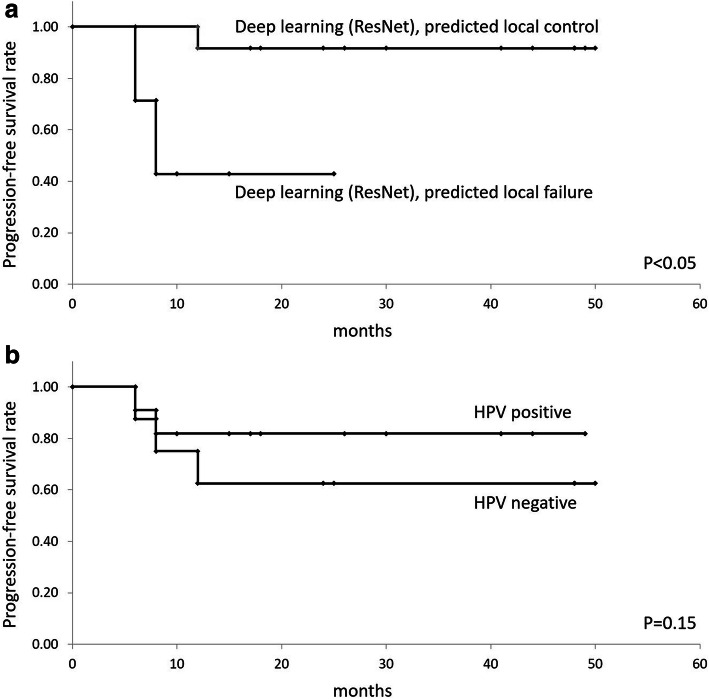
Results of the Kaplan-Meier analyses in subgroup analysis based on HPV status. The local PFS rate was significantly greater in low risk of deep learning-based classification with axial and coronal images combination model in ResNet (**A**) (*P* < .05). The local PFS rate was visually divided in the Kaplan-Meier curve by HPV status-based classification (**B**). However, there was no statistical significance (*P* = 0.15)

As subgroup analysis based on the HPV status, HPV status of a total of 19 patients in the test set were available and assessed their PFS rate; the local PFS rate was significantly greater using deep learning-based classification (*P* < .05), whereas the local PFS rate could be visually divided in the Kaplan-Meier curve by HPV status-based classification, however, statistical significance was not observed (*P* = 0.15) (Fig.5). In further subgroup analysis with the division of HPV positive (*n* = 11) and negative status (*n* = 8) in test set cohorts, the Kaplan-Meier curve analysis provided the division of local PFS rate more clearly in deep learning classification compared to a multivariate clinical model (Suppl Fig. 1).

In the assessment of the robustness of deep learning classification models created by partly switching training with test set cohorts, the diagnostic performance of axial and coronal combination model in ResNet using the test set consisting of A-1 group was as follows; sensitivity 0.78, specificity 0.83, PPV 0.89, NPV 0.68, and accuracy 0.80. In contrast, diagnostic performance of test set consisting of A-2 group was as follows; sensitivity 0.83, specificity 0.80, PPV 0.90, NPV 0.67, and accuracy 0.82. The Kaplan-Meier curve showed a clear division of PFS rate in both classification models (Suppl Fig. 2).

## Discussion

Our analysis demonstrates that it is possible to generate a deep learning-based model using FDG-PET to predict the treatment outcome of OPSCCs. This deep learning diagnostic model showed high diagnostic accuracy for predicting treatment outcome and local PFS in the test dataset. It was superior to the T-stage and clinical stage classification, which is widely used in current clinical practice to assess the pretreatment prognosis. Notably, the multi-dimensional image approach with the ResNet architecture provided the highest diagnostic accuracy. This demonstrates the potential of this architecture as a clinical tool. To the best of our knowledge, this study was the first report which investigated the utility of deep learning analysis for the prediction of treatment outcome targeting patients with OPSCCs.

In the clinical setting, the local treatment outcome’s prediction has a significant impact on clinical management. If a poor outcome after chemoradiation therapy is predicted, additional or alternate treatment plans such as induction chemotherapy and earlier salvage surgery or additional chemotherapy/immunotherapy after chemoradiotherapy may be offered. Follow-up strategy after the treatment, such as the frequency and duration of the imaging follow-up, may also be optimized individually, depending on the pretreatment risks.

Imaging findings of FDG-PET to predict the treatment outcome in patients with OPSCC have been described in several past reports. The utility of conventional FDG-PET derived parameters such as SUVmax, SUVmean, MTV, and TLG was firstly investigated in several studies [5–7]; higher SUVmax and SUVmean, larger MTV and TLG were respectively described as being associated with poor prognosis. In recent years, with the development of texture analysis in the field of radiology, the utility of intratumoral heterogeneity in the primary tumor was reported using texture analysis of FDG-PET and CT [9–11, 21]. In these studies, an increase of intratumoral heterogeneity in the primary site indicated a poor prognosis in OPSCC patients. Such intratumoral characteristics of heterogeneity might reflect the tumor biological difference of sensitivity to chemoradiation therapy. In addition, morphological information of primary tumor shape outlined by SUV uptake area was described as one of the prognostic factors in head and neck cancer patients; complicated tumor shape such as ‘larger tumor asphericity’ or ‘greater tumor irregularity’ detected by imaging are associated treatment failure [8, 9, 22]. These findings hint at tumor features that are not captured by the popular T-staging system. Deep learning algorithms are most suited to identify these features and generate an optimal diagnostic model by associating these imaging characteristics with treatment outcomes. More recently, the radiomics approach has flourished with new developments in image analysis. With the latest methods, imaging characteristics can be selected and associated with radiomics signature to identify parameters useful for the diagnosis and prognosis; diagnostic performance obtained with this technique was indicated superior to a simple use of the aforementioned parameters (e.g., SUVmax, SUVmean, MTV, TLG, intratumoral texture, tumor asphericity and irregularity) [23]. Although the diagnostic power of deep learning vs. radiomics analysis was not directly compared in the current study, others have reported on radiomics analysis to assess OPSCCs. These studies inform us of the diagnostic power associated with radiomic analysis [23–27]. Notably, two studies assessed FDG-PET image-based radiomics analysis and reported an AUC of around 0.7 for predicting local failure [23, 24]. Our AUC for deep learning analysis compares favorably with the radiomics report. In addition, deep learning analysis can be performed without complicated manual calculation processes such as tumor outline delineation or intratumoral heterogeneity measurement performed in radiomics analysis. This could be one of the major advantages of a deep learning-based diagnostic model. Although the CNN might identify imaging features and metabolic characteristics (e.g., degree of SUV uptake, intratumoral heterogeneity of FDG uptake, the borders of the primary tumor, type of tumor shape, and extension of the primary tumor, etc.) and integrate these features to discriminate between good and poor treatment outcome, however, actually selected features/characteristics for the model creation were hidden and was considered in black-box, this was one of the great limitations in deep learning analysis. Future analysis with a new algorithm that clarifies the internal decision process of deep learning analysis will be needed.

In the current study, three architectures of AlexNet, GoogleNet, and ResNet were used for deep learning analysis. ResNet was revealed to have the highest AUC among the three architectures, whereas, GoogleNet also indicated to have high AUC and not much different from ResNet. However, a lower AUC compared to the aforementioned two architectures was shown in AlexNet. AlexNet consists of eight layers; this was so simple structure compared to GoogLeNet and ResNet. The difference in diagnostic accuracy might be because of such structural differences among these three architectures.

The current study has several limitations. First, FDG-PET/CT scanners were different for the training and test cohorts. In addition, all patients received chemoradiation therapy with curative intent as the main treatment method. However, quite a few patients received induction chemotherapy before the main treatment. Some patients also received surgical neck resection after the main treatment. Furthermore, the distribution of patient numbers was not well balanced among T1–4 groups (mildly biased in T2 and T3) and N1–3 groups (also mildly biased in N2b and N2c). Because deep learning analysis generally needs a large sample size to create a sufficiently learned model, it might be challenging to collect a completely homogeneous cohort with imaging from only one scanner and with the same treatments. Next, the status of human papillomavirus (HPV) in many patients was unknown because OPSCC patients diagnosed in this study span 10 years (2007–2017) when not all patients were tested for the presence of the virus. HPV status is now known to be an independent prognostic factor in OPSCCs [28]. Although a potential of high diagnostic performance in the deep learning model and its independence from the HPV status might be indicated by results from the subgroup analysis in the current study, further analyses with the division of total patients into homogeneous treatment regimens and positive/negative HPV status are needed to address these limitations. In addition, the previous investigation described the diagnostic model to predict the HPV status from deep learning-based image analysis using FDG-PET images [14]. The integrated use, including this previously described model, might contribute more accurate diagnosis in deep learning-based local prognosis prediction as a future analysis.

## Conclusions

A deep learning-based diagnostic model using FDG-PET images can potentially predict treatment outcome and local progression-free survival rate in patients with OPSCC who received definitive chemoradiation therapy.

## Supplementary Information

**Additional file 1: Figure S1.** Results of the Kaplan-Meier curve analysis in HPV positive and negative group. The Kaplan-Meier curve by deep learning-based classification with axial and coronal images combination model in ResNet and the multivariate clinical model in HPV positive patients group (A, B) and in HPV negative patients group (C, D) were presented respectively.

**Additional file 2: Figure S2.** Results of the Kaplan-Meier curve analyses with switching patients cohort between training and test session. The Kaplan-Meier curve of axial and coronal combination model in ResNet using test set consisting of A-1 group (A) and A-2 group (B) was presented. A clear division of PFS rate in both Kaplan-Meier curves was observed.

## Data Availability

The datasets used and/or analyzed during the current study are available from the corresponding author on reasonable request.

## Abbreviations

AUC: Area under curve
CNN: Convolutional neural network
HPV: Human papillomavirus
JPEG: Joint Photographic Experts Group
MTV: Metabolic tumor volume
OPSCC: Oropharyngeal squamous cell carcinoma
PFS: Progression-free survival
ROC: Receiver operating characteristic
SUV: Standardized uptake value
TLG: Total lesion glycolysis
