# Supplementary figures and images for: Prediction of the local treatment outcome in patients with oropharyngeal squamous cell carcinoma using deep learning analysis of pretreatment FDG-PET images
Source: BMC Cancer. 2021 Aug 6;21:900. doi: 10.1186/s12885-021-08599-6 (PMC8344209; doi:10.1186/s12885-021-08599-6)

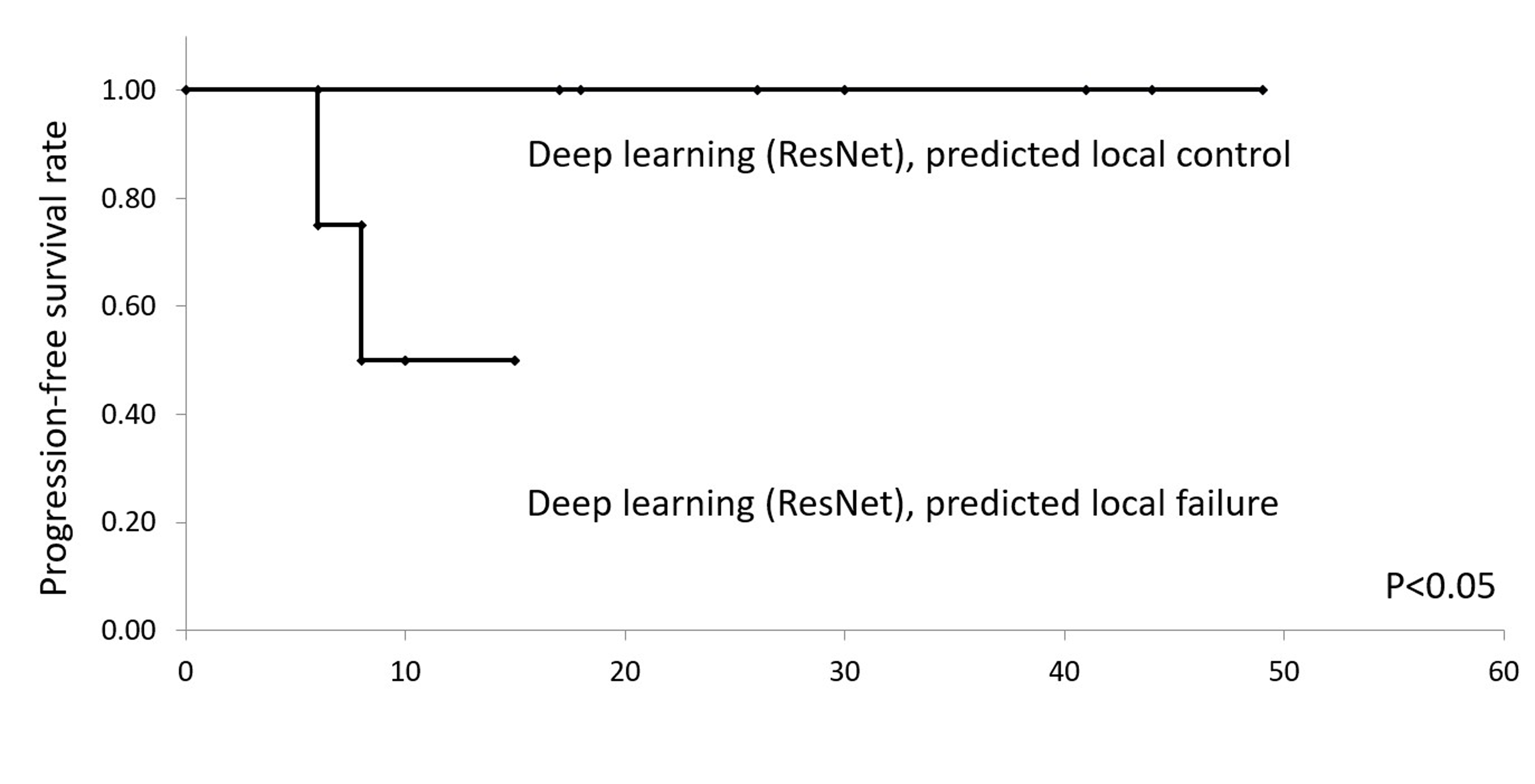

Supplement: Supplementary file 1 — Additional file 1: Figure S1. Results of the Kaplan-Meier curve analysis in HPV positive and negative group. The Kaplan-Meier curve by deep learning-based classification with axial and coronal images combination model in ResNet and the multivariate clinical model in HPV positive patients group (A, B) and in HPV negative patients group (C, D) were presented respectively. [file 12885_2021_8599_MOESM1_ESM.zip › Suppl Fig1aR3.tif]

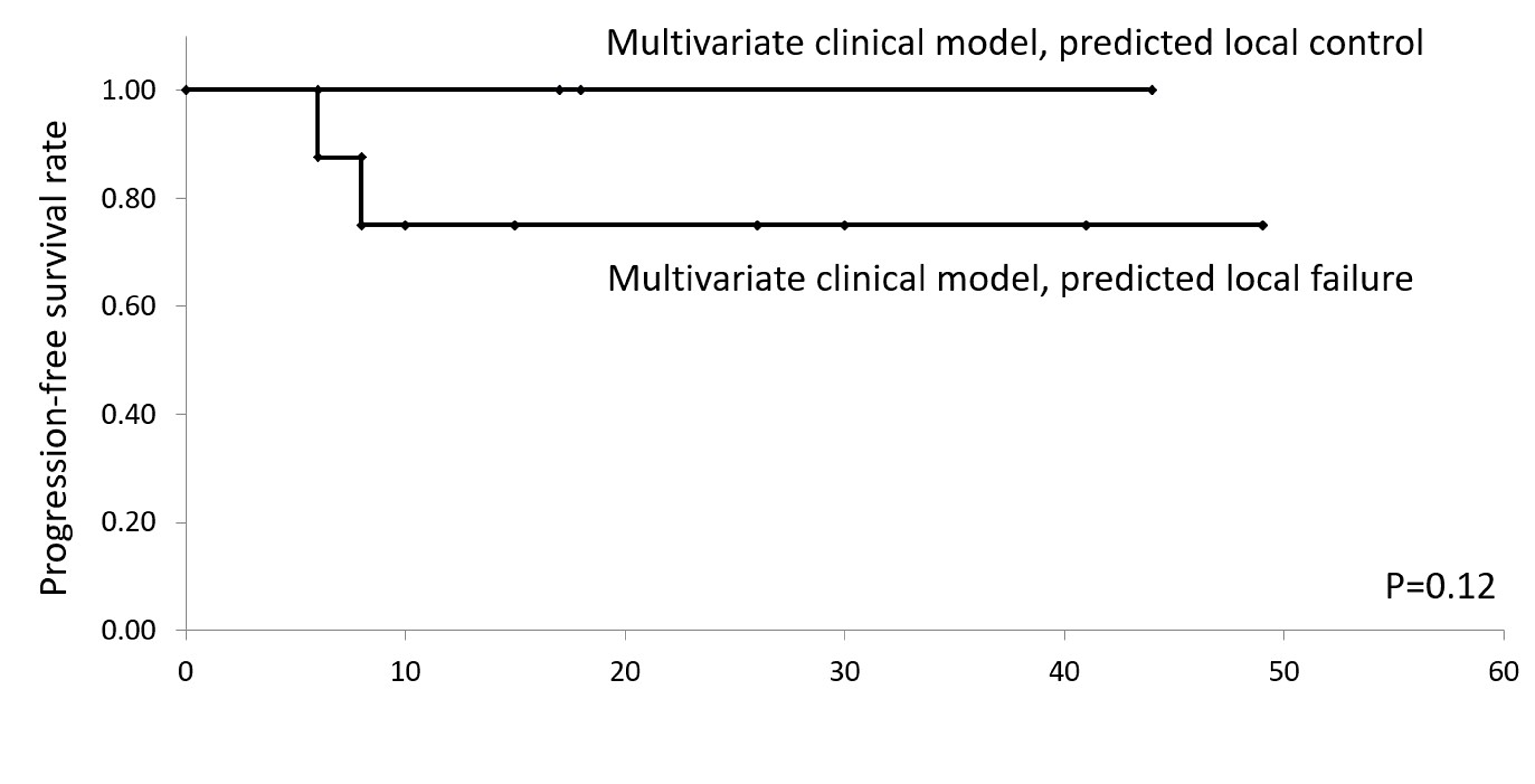

Supplement: Supplementary file 1 — Additional file 1: Figure S1. Results of the Kaplan-Meier curve analysis in HPV positive and negative group. The Kaplan-Meier curve by deep learning-based classification with axial and coronal images combination model in ResNet and the multivariate clinical model in HPV positive patients group (A, B) and in HPV negative patients group (C, D) were presented respectively. [file 12885_2021_8599_MOESM1_ESM.zip › Suppl Fig1bR3.tif]

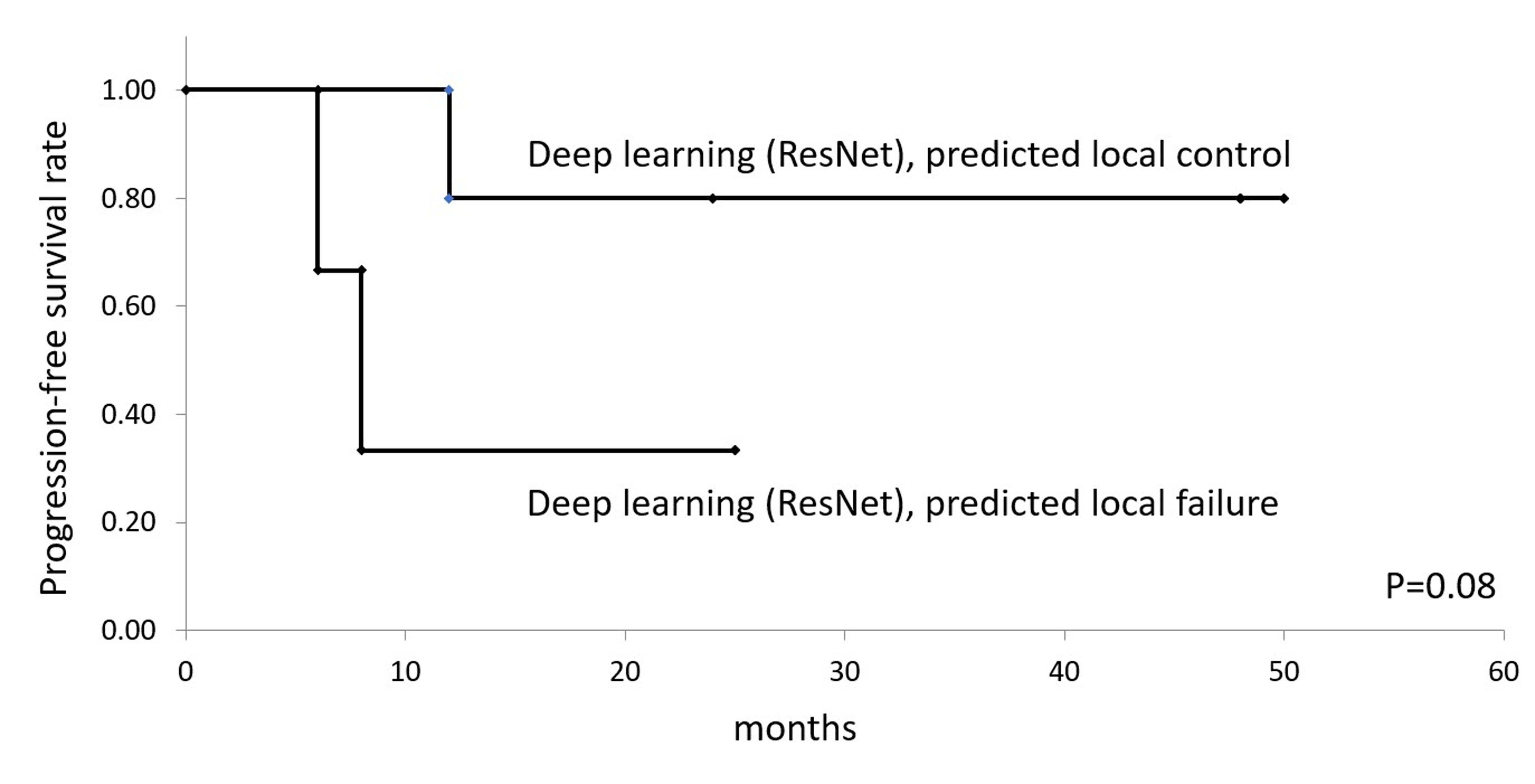

Supplement: Supplementary file 1 — Additional file 1: Figure S1. Results of the Kaplan-Meier curve analysis in HPV positive and negative group. The Kaplan-Meier curve by deep learning-based classification with axial and coronal images combination model in ResNet and the multivariate clinical model in HPV positive patients group (A, B) and in HPV negative patients group (C, D) were presented respectively. [file 12885_2021_8599_MOESM1_ESM.zip › Suppl Fig1cR3.tif]

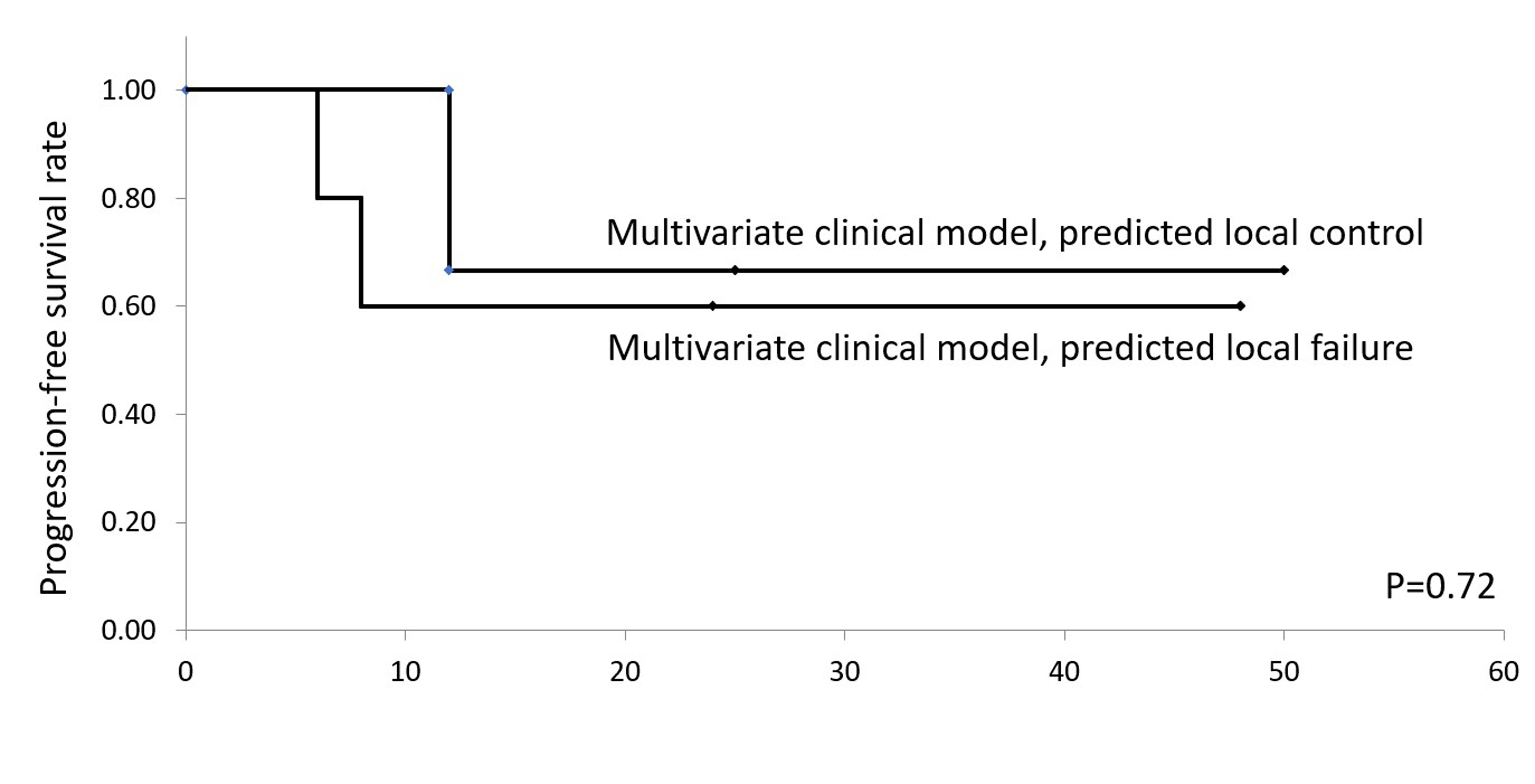

Supplement: Supplementary file 1 — Additional file 1: Figure S1. Results of the Kaplan-Meier curve analysis in HPV positive and negative group. The Kaplan-Meier curve by deep learning-based classification with axial and coronal images combination model in ResNet and the multivariate clinical model in HPV positive patients group (A, B) and in HPV negative patients group (C, D) were presented respectively. [file 12885_2021_8599_MOESM1_ESM.zip › Suppl Fig1dR3.tif]

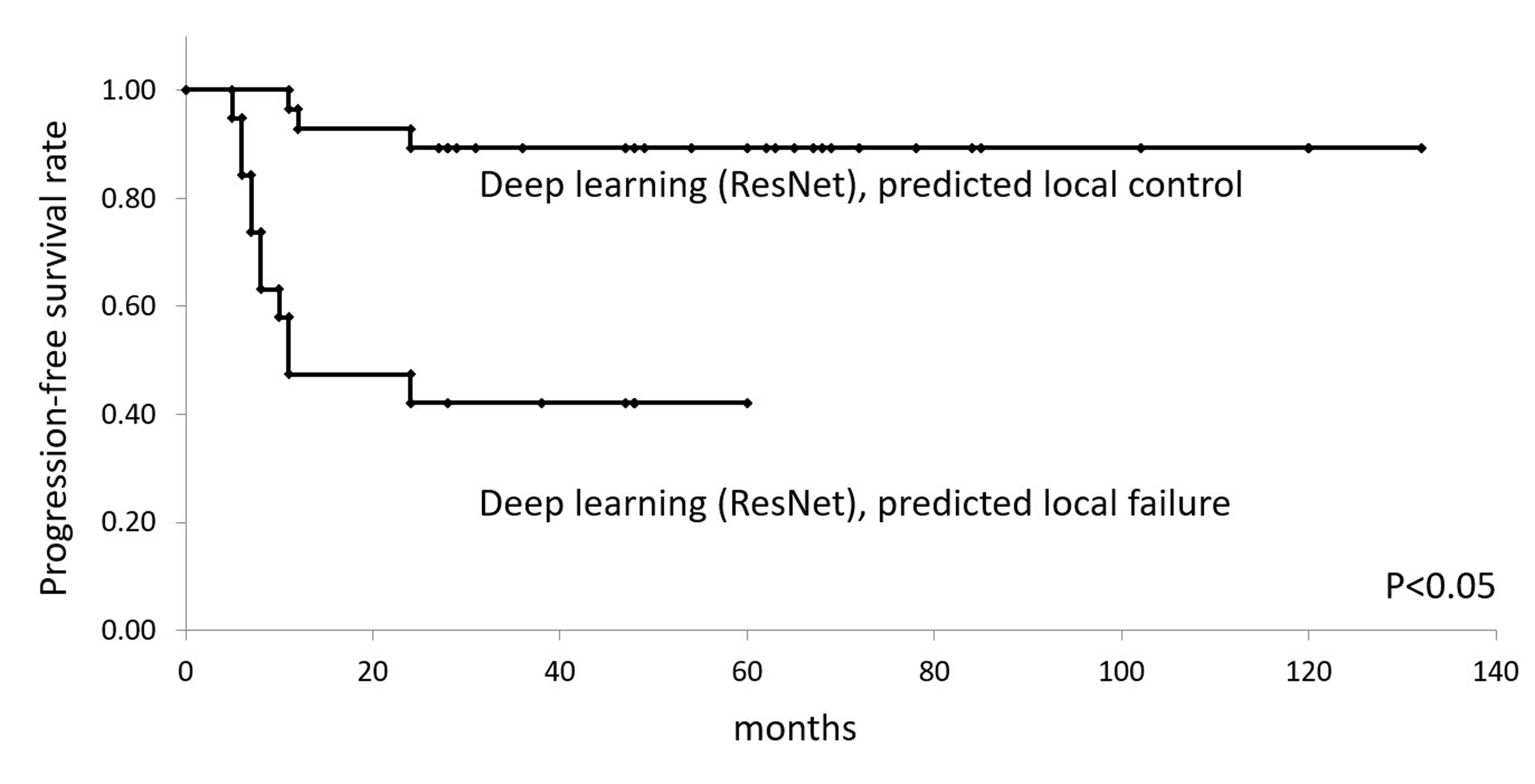

Supplement: Supplementary file 2 — Additional file 2: Figure S2. Results of the Kaplan-Meier curve analyses with switching patients cohort between training and test session. The Kaplan-Meier curve of axial and coronal combination model in ResNet using test set consisting of A-1 group (A) and A-2 group (B) was presented. A clear division of PFS rate in both Kaplan-Meier curves was observed. [file 12885_2021_8599_MOESM2_ESM.zip › Suppl Fig2aR3.tif]

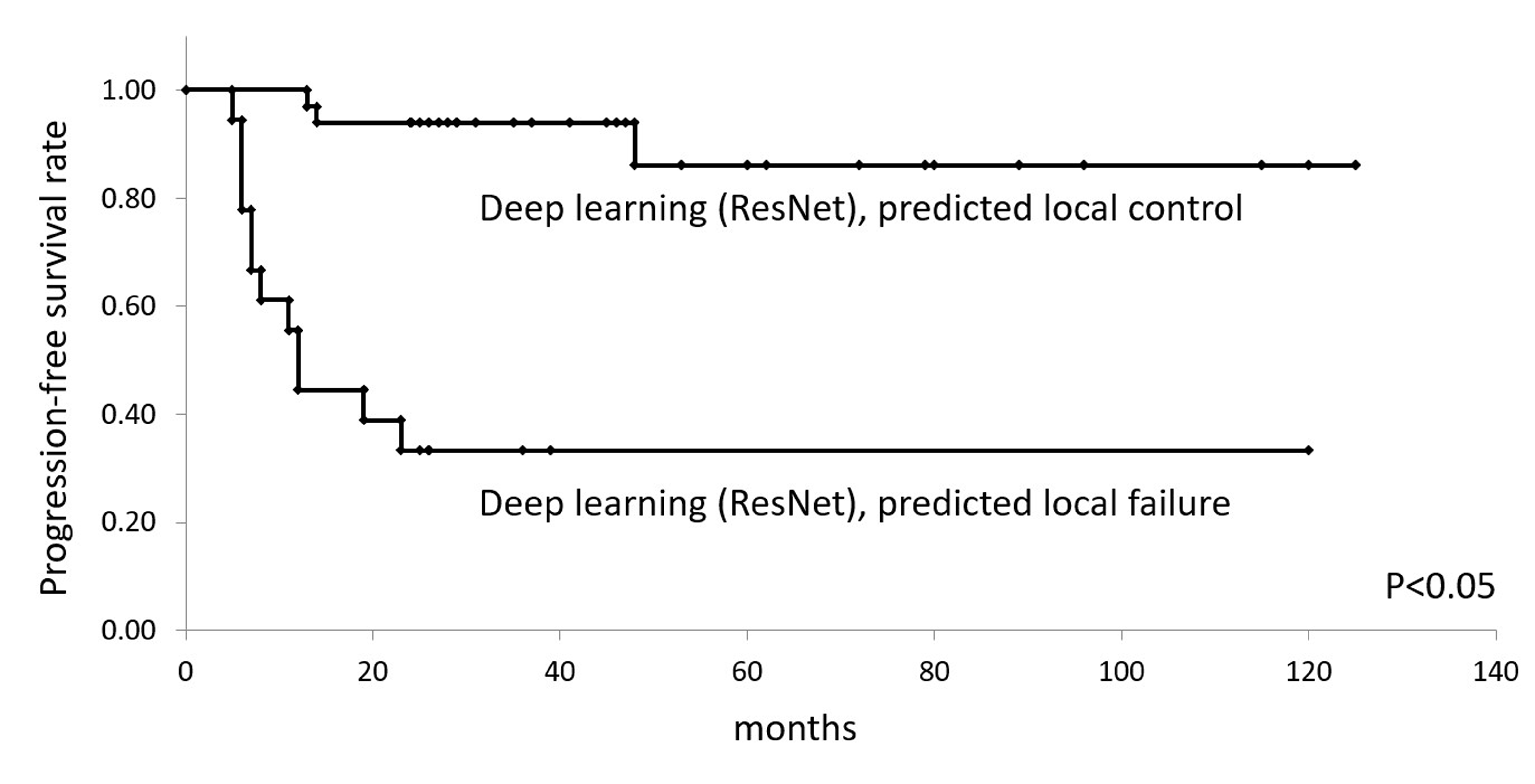

Supplement: Supplementary file 2 — Additional file 2: Figure S2. Results of the Kaplan-Meier curve analyses with switching patients cohort between training and test session. The Kaplan-Meier curve of axial and coronal combination model in ResNet using test set consisting of A-1 group (A) and A-2 group (B) was presented. A clear division of PFS rate in both Kaplan-Meier curves was observed. [file 12885_2021_8599_MOESM2_ESM.zip › Suppl Fig2bR3.tif]
